# Supplementary material for: Identification of Potential Phytochemical/Antimicrobial Agents against Pseudoperonospora cubensis Causing Downy Mildew in Cucumber through In-Silico Docking
Source: Plants (Basel). 2023 Jun 2;12(11):2202. doi: 10.3390/plants12112202 (PMC10255482; doi:10.3390/plants12112202)
Supplement: Supplementary file 1 [file plants-12-02202-s001.zip › Supplementary Figure S1.pdf]

Supplementary Figure S1. 3D visualization of the interaction between QNE4 effector protein with top nine phytochemicals A) Cucumerin-A B) Cucumerin-B C) Isoscoparin D) Apigenin -7-O –glucoside .E) Cucurbitacin-B F) Cucurbitacin-D G) Cucurbitacin-A H) Cucurbitacin-E I) Cucurbitacin-I

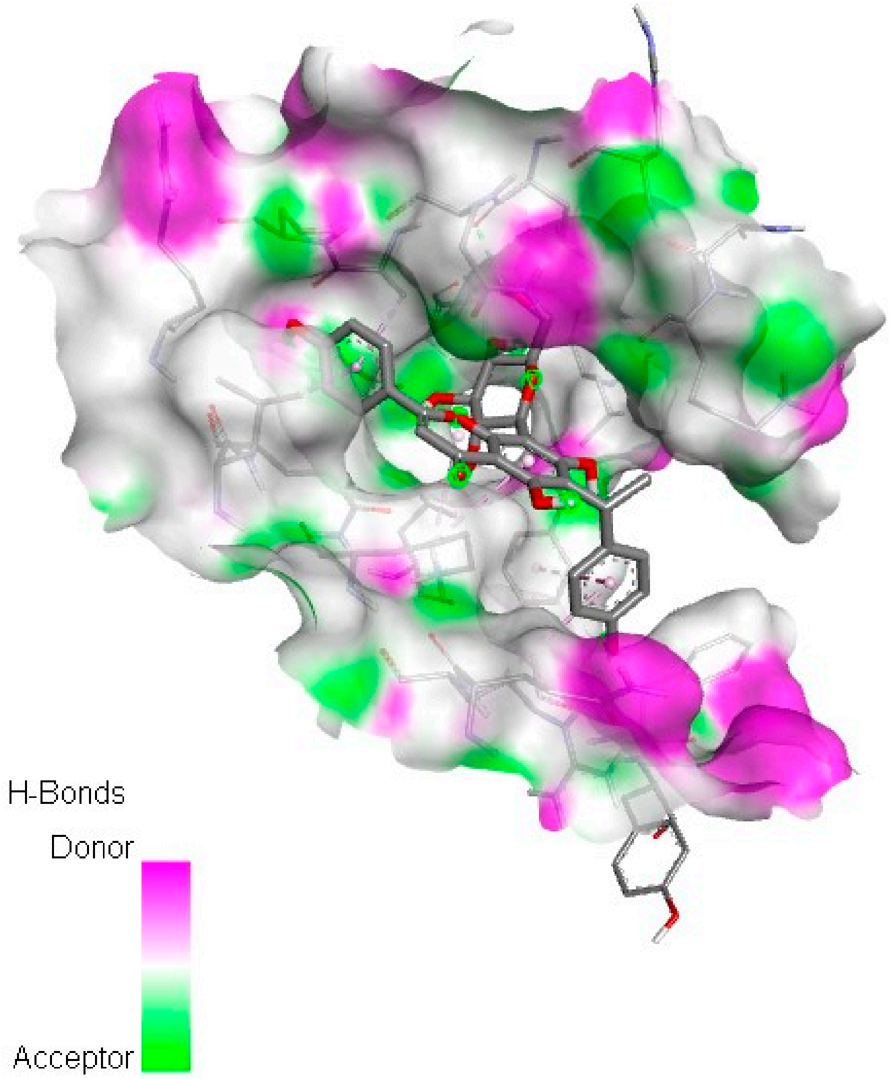

A) Cucumerin-A

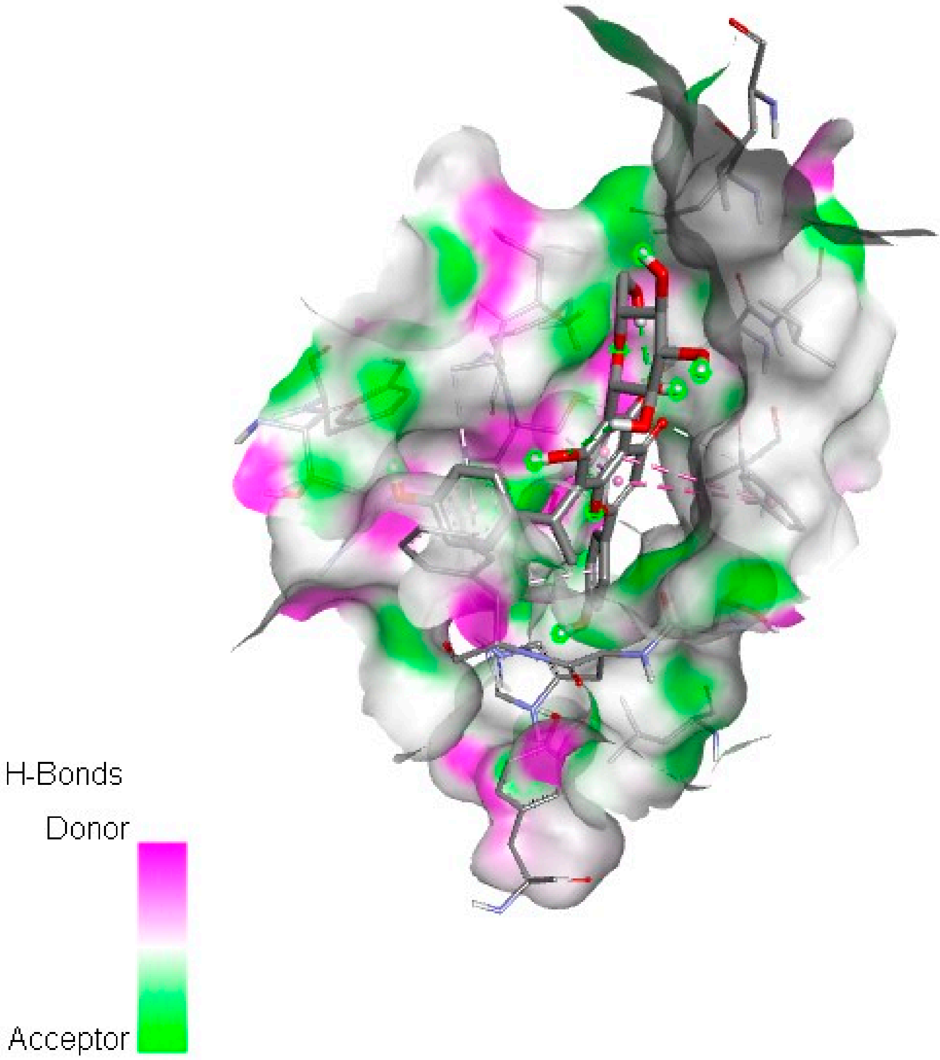

B) Cucumerin-B

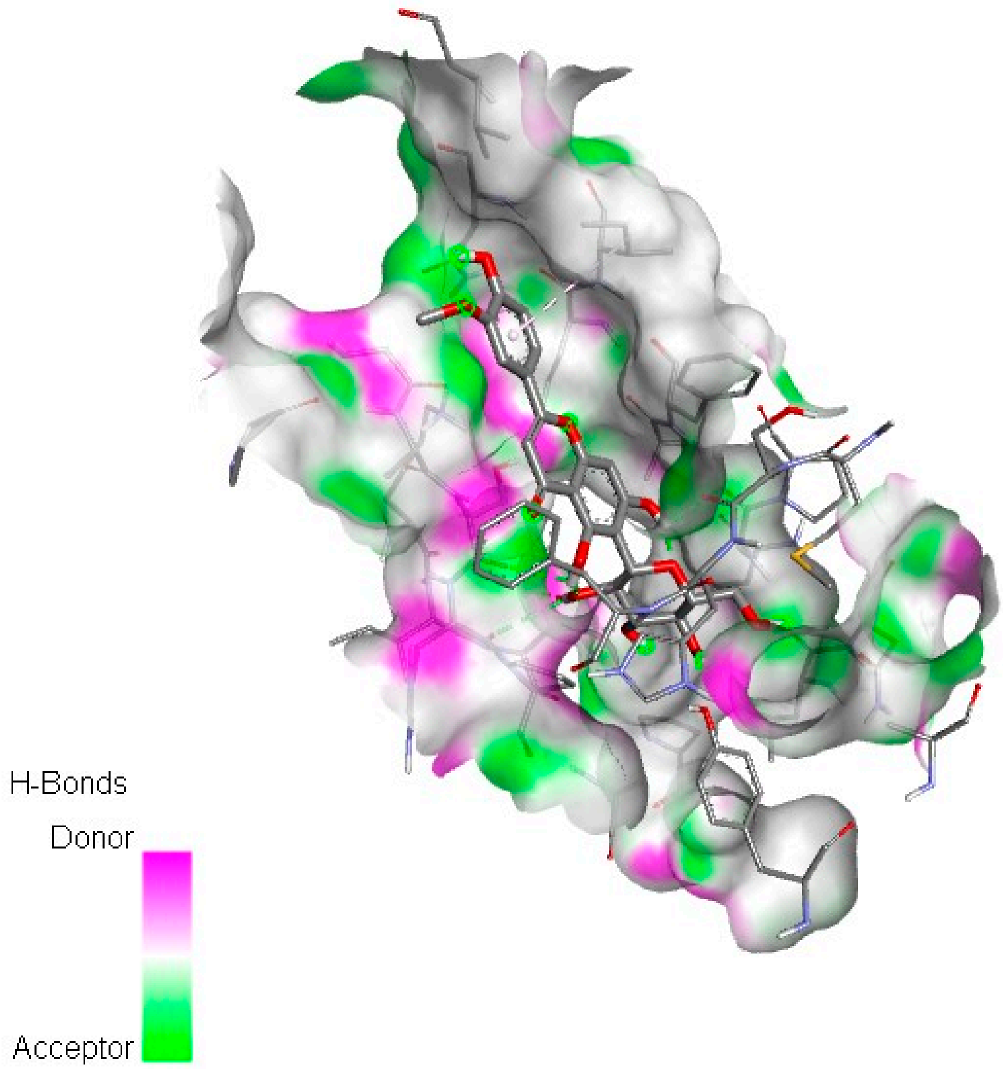

C) Isoscoparin

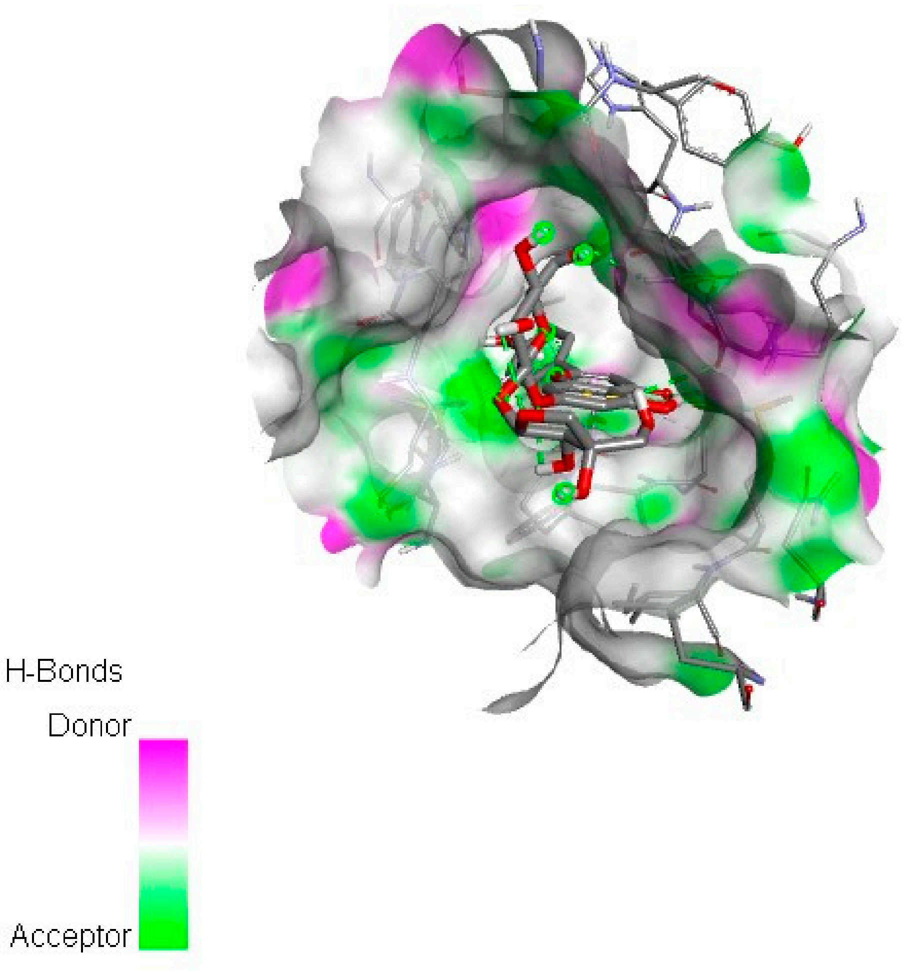

D) Apigenin -7-O –glucoside

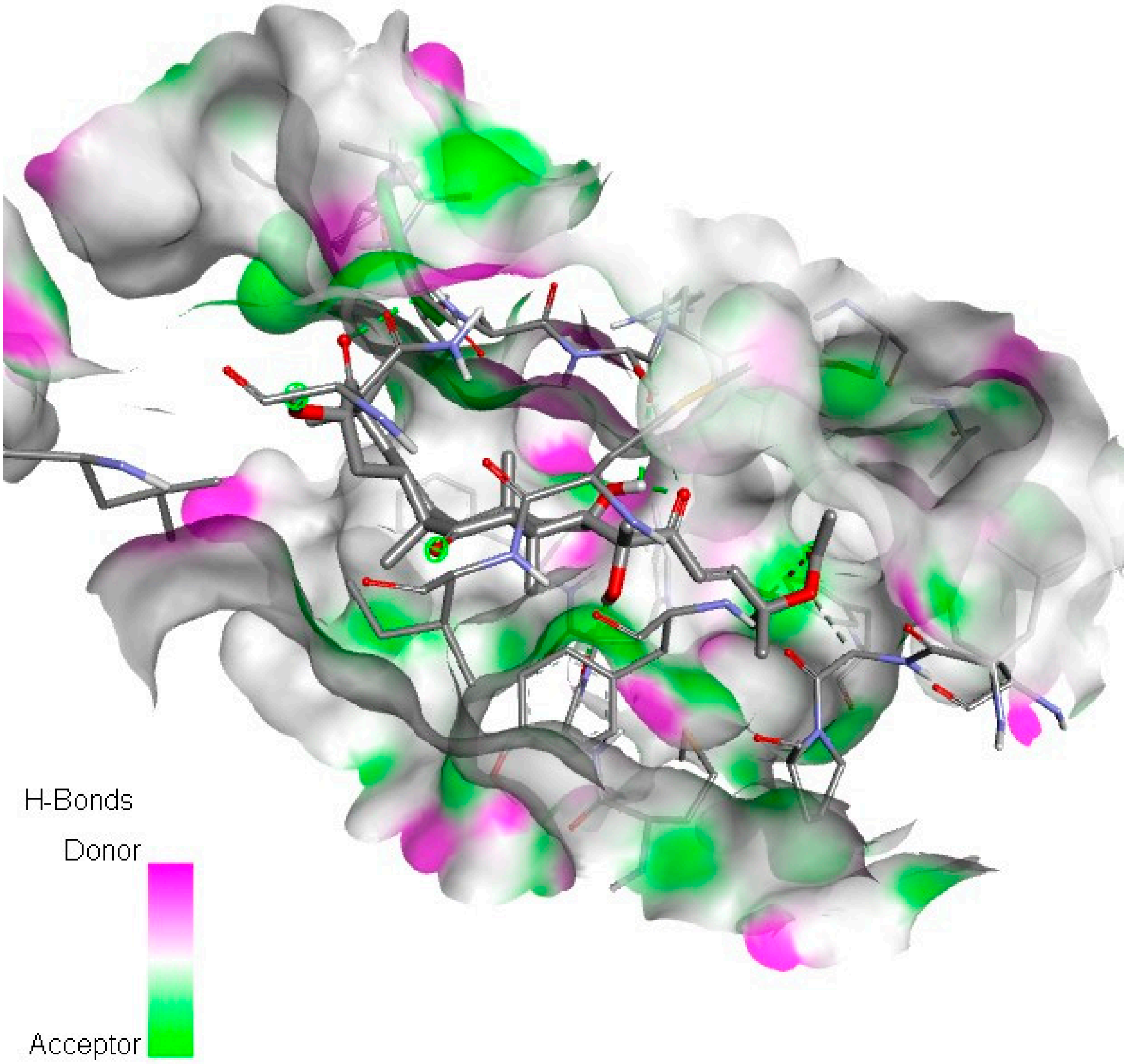

E) Cucurbitacin-B

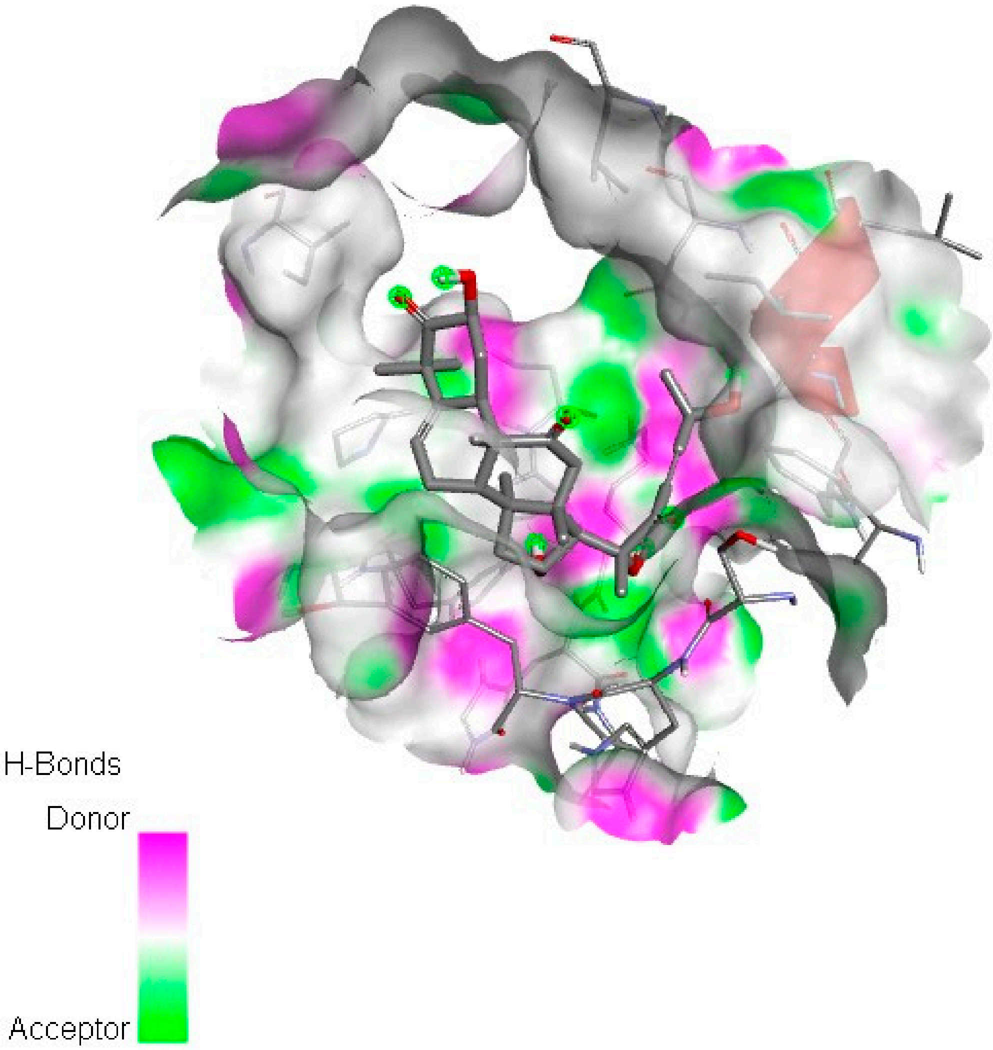

F) Cucurbitacin-D

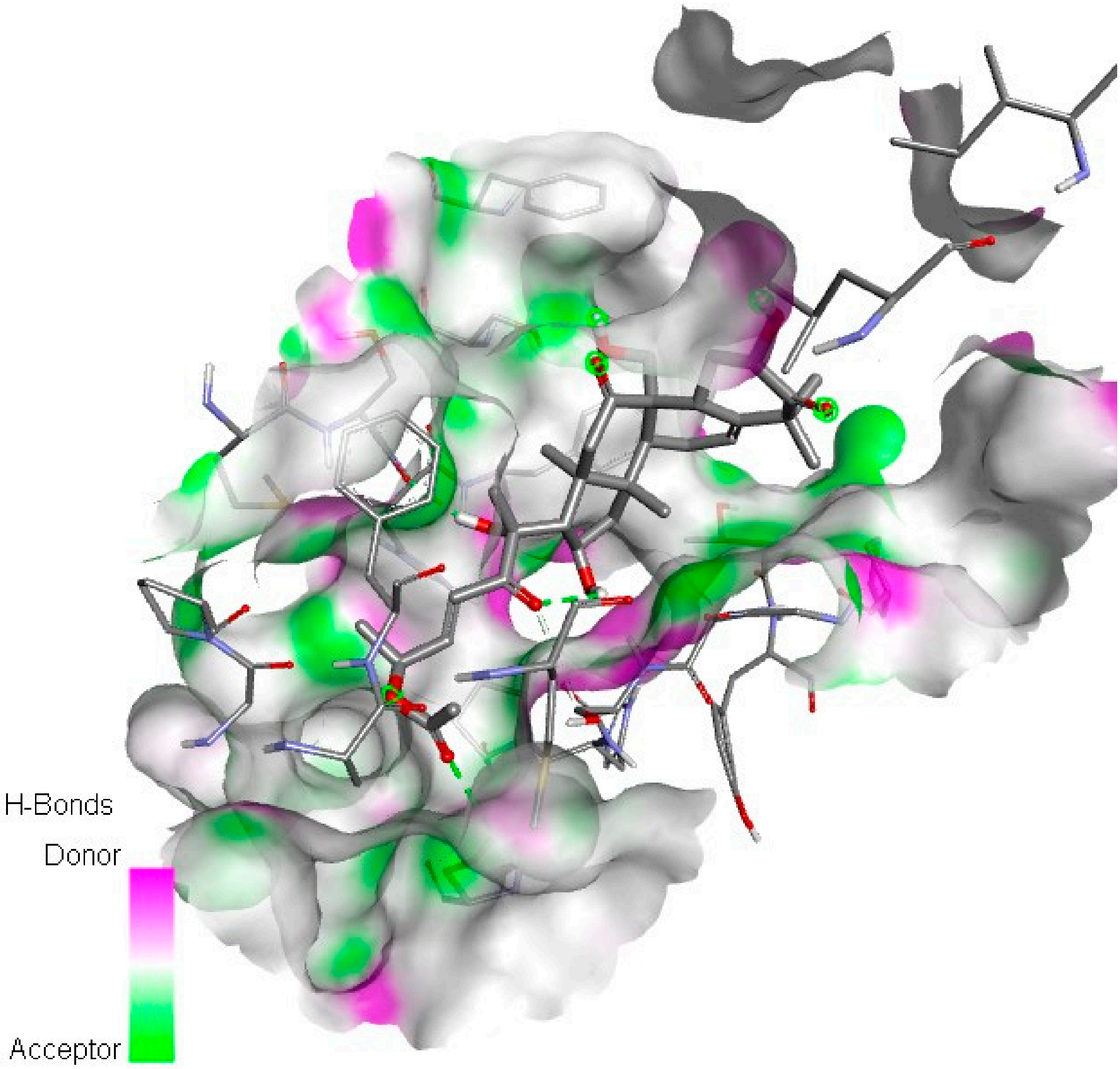

G) Cucurbitacin-A

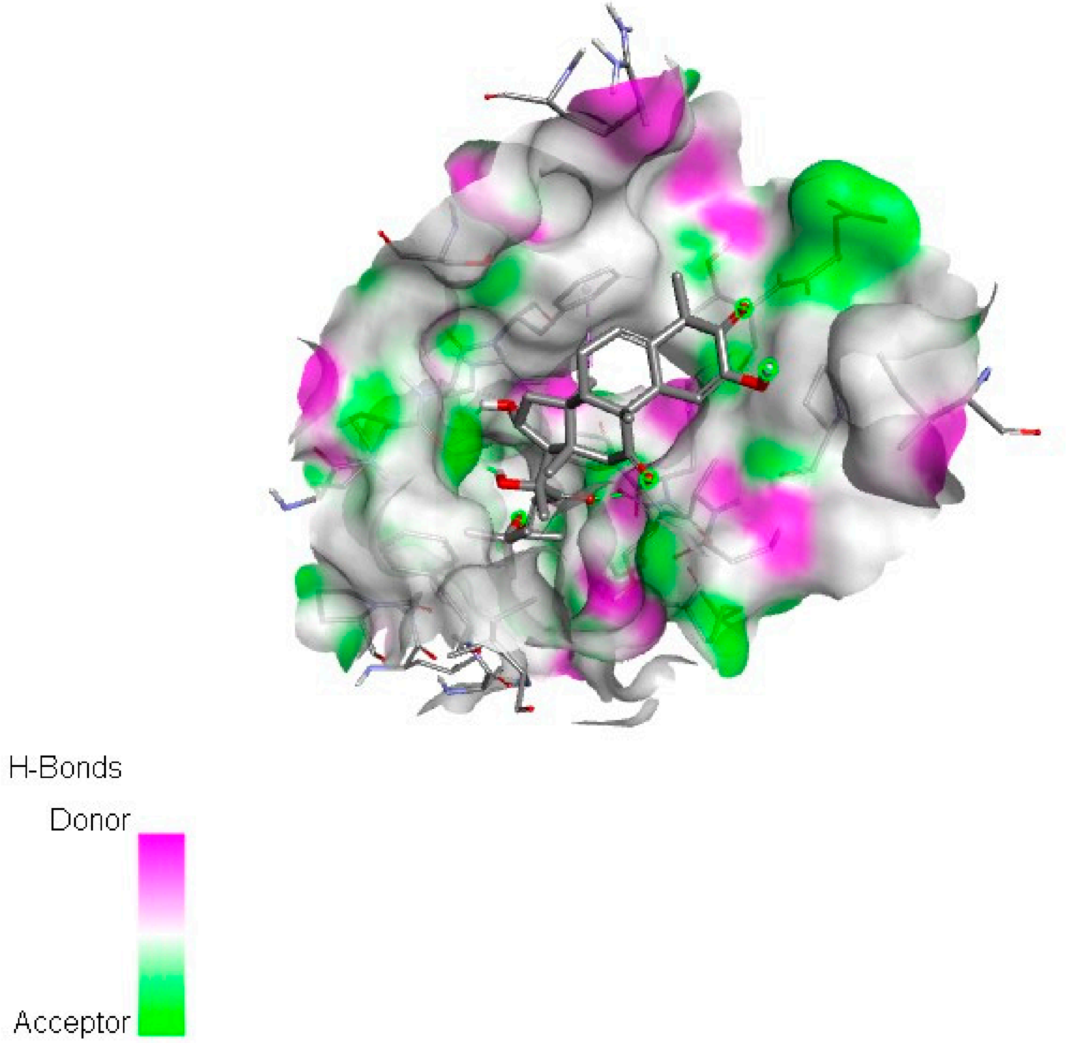

H) Cucurbitacin-E

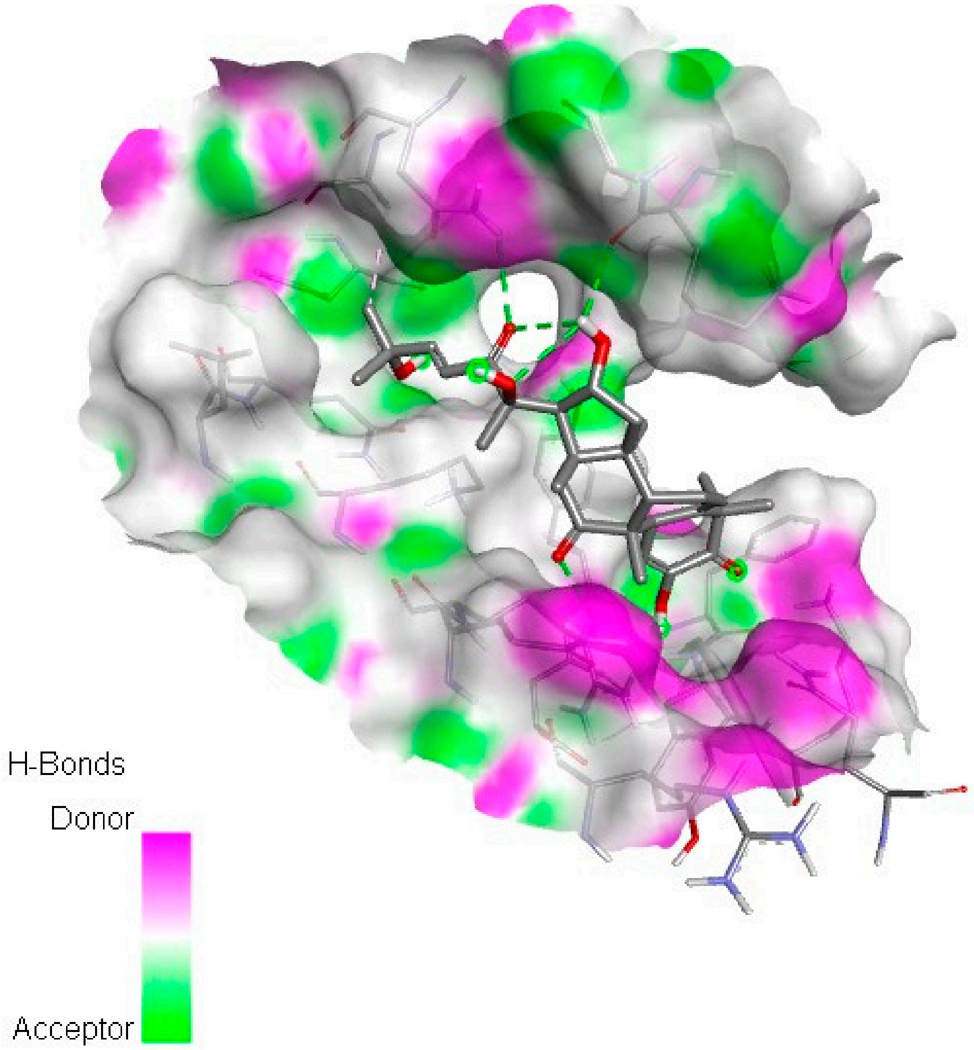

I) Cucurbitacin-I
