# Supplementary material for: Identification of Potential Phytochemical/Antimicrobial Agents against Pseudoperonospora cubensis Causing Downy Mildew in Cucumber through In-Silico Docking
Source: Plants (Basel). 2023 Jun 2;12(11):2202. doi: 10.3390/plants12112202 (PMC10255482; doi:10.3390/plants12112202)
Supplement: Supplementary file 1 [file plants-12-02202-s001.zip › Supplementary Figure S3.pdf]

**Supplementary Figure S3.** 3D visualization of the interaction between QNE 4 effector protein with top compounds from botanicals and chemical sources A) Azoxystrobin B) Allyl acetate C) Salicylic acid D) Curzate E) Allixin

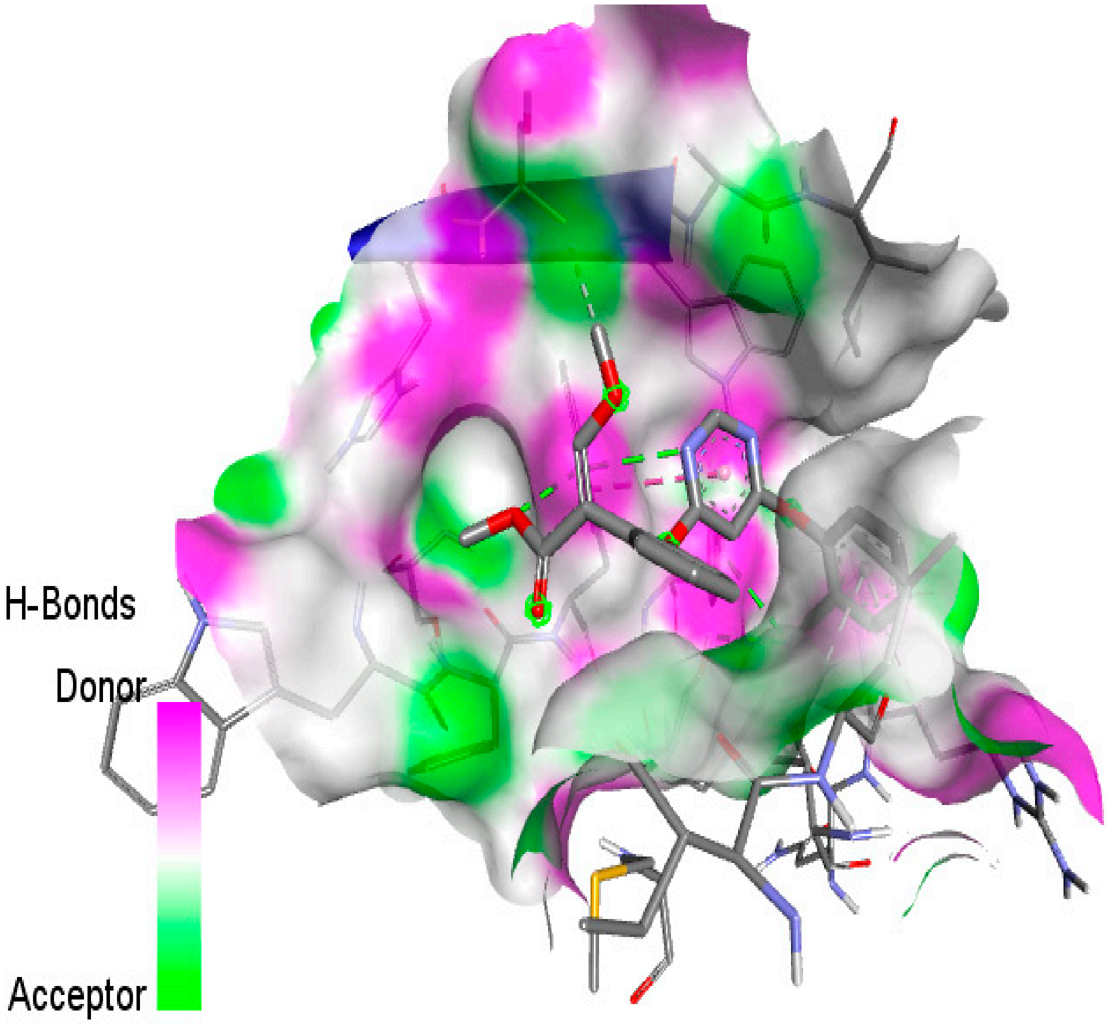


**A) Azoxystrobin**

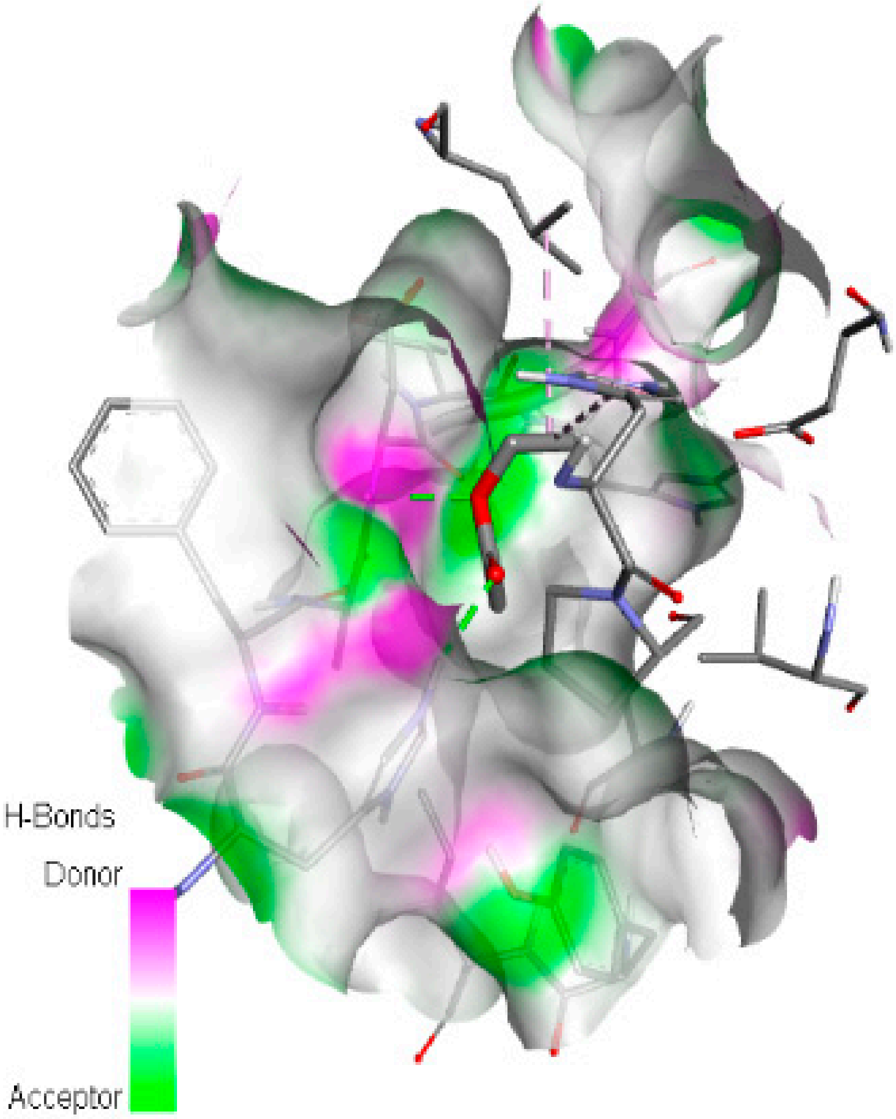


**B) Allyl acetate**

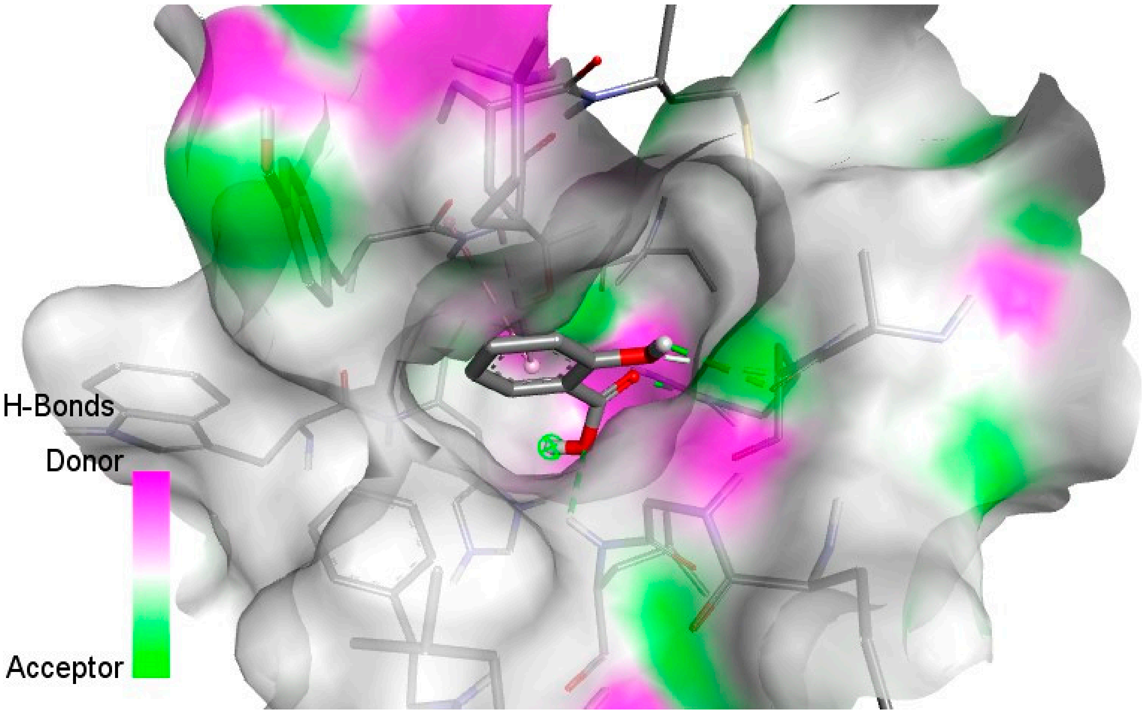


**C) Salicylic acid**

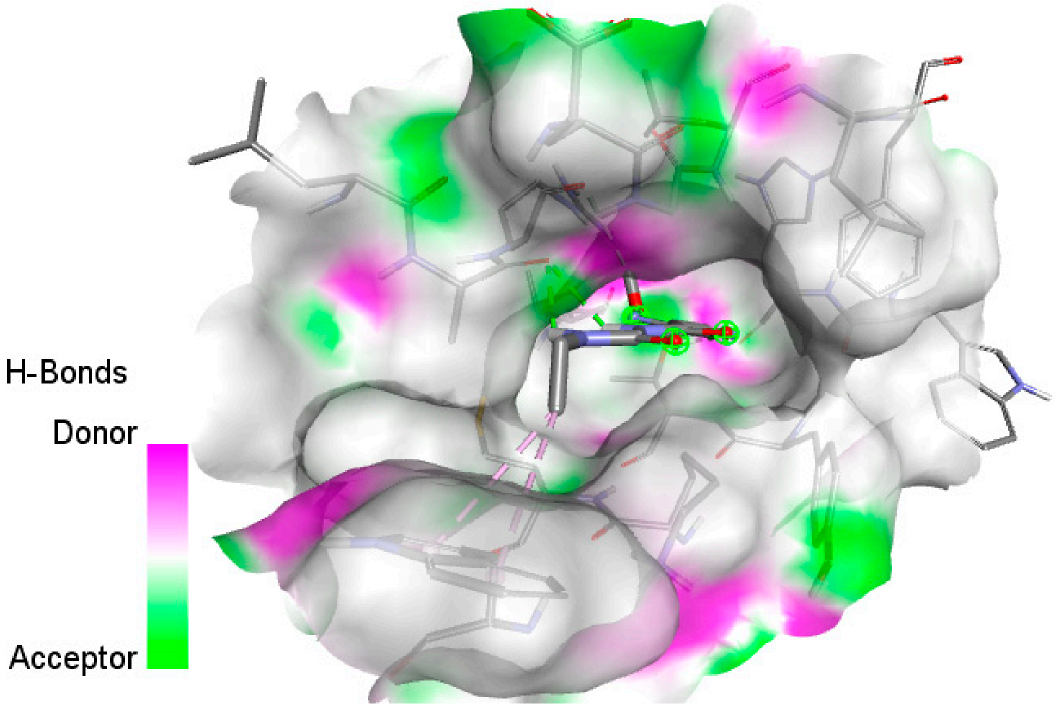


**D) Curzate**

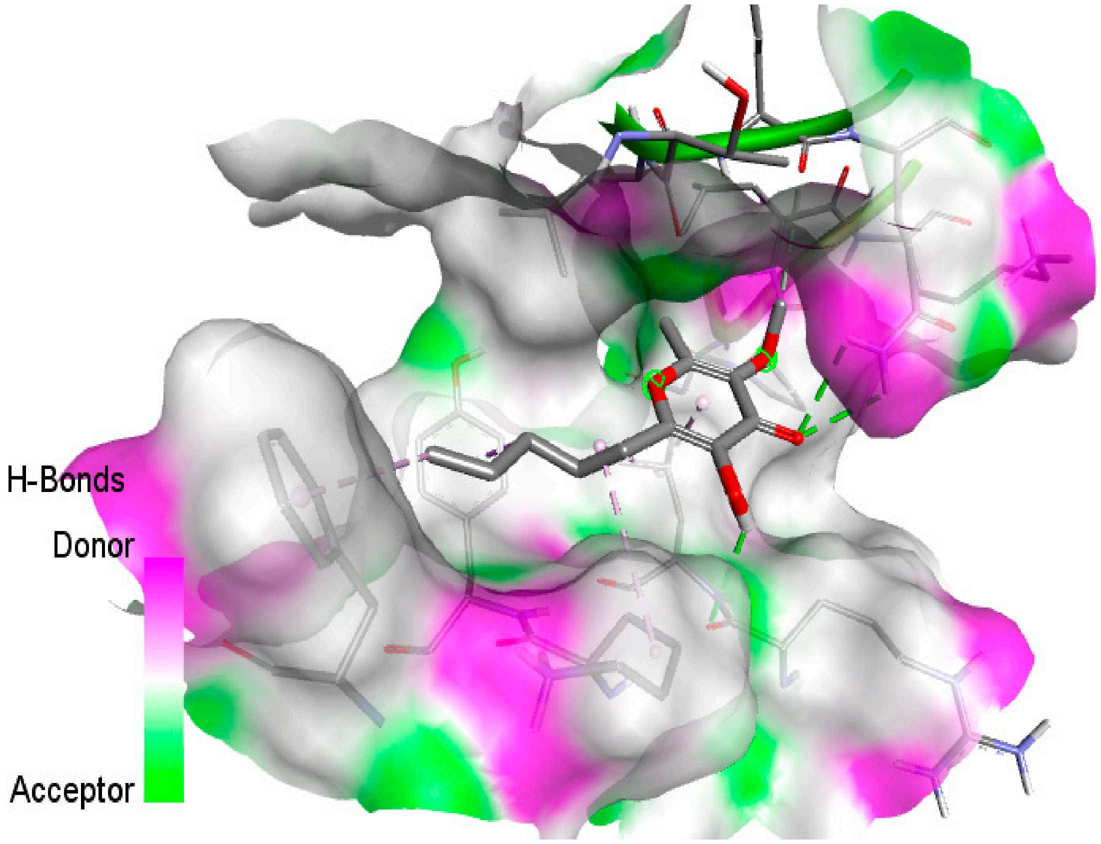


**E) Allixin**
