# Supplementary material for: Identification of Potential Phytochemical/Antimicrobial Agents against Pseudoperonospora cubensis Causing Downy Mildew in Cucumber through In-Silico Docking
Source: Plants (Basel). 2023 Jun 2;12(11):2202. doi: 10.3390/plants12112202 (PMC10255482; doi:10.3390/plants12112202)
Supplement: Supplementary file 1 [file plants-12-02202-s001.zip › Supplementary Figure S4.pdf]

Supplementary Figure S4. 3D visualization of the interaction between cytochrome oxidase subunit 1 protein with top nine compounds from botanicals and chemical sources A) Azoxystrobin B) Allyl acetate C) Kresoxim methyl D) Curzate.

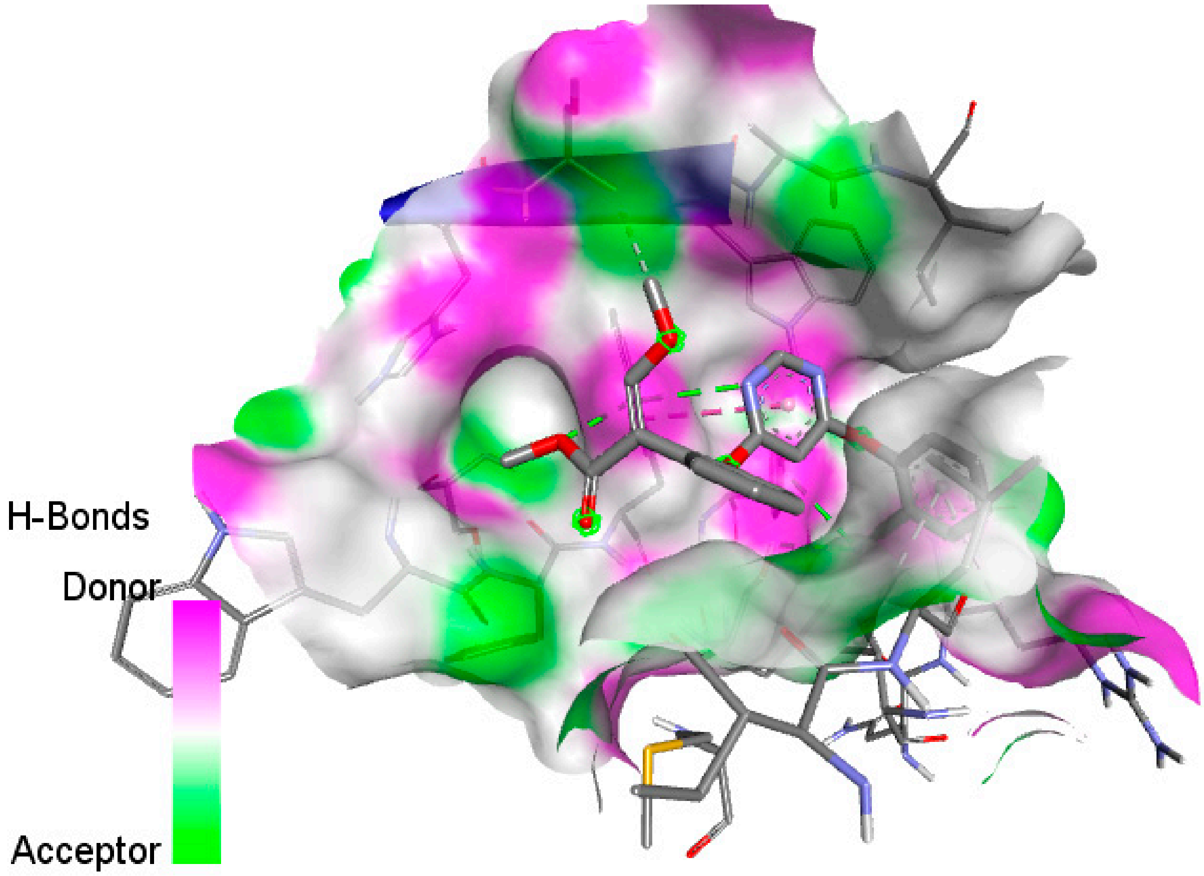

A) Azoxystrobin

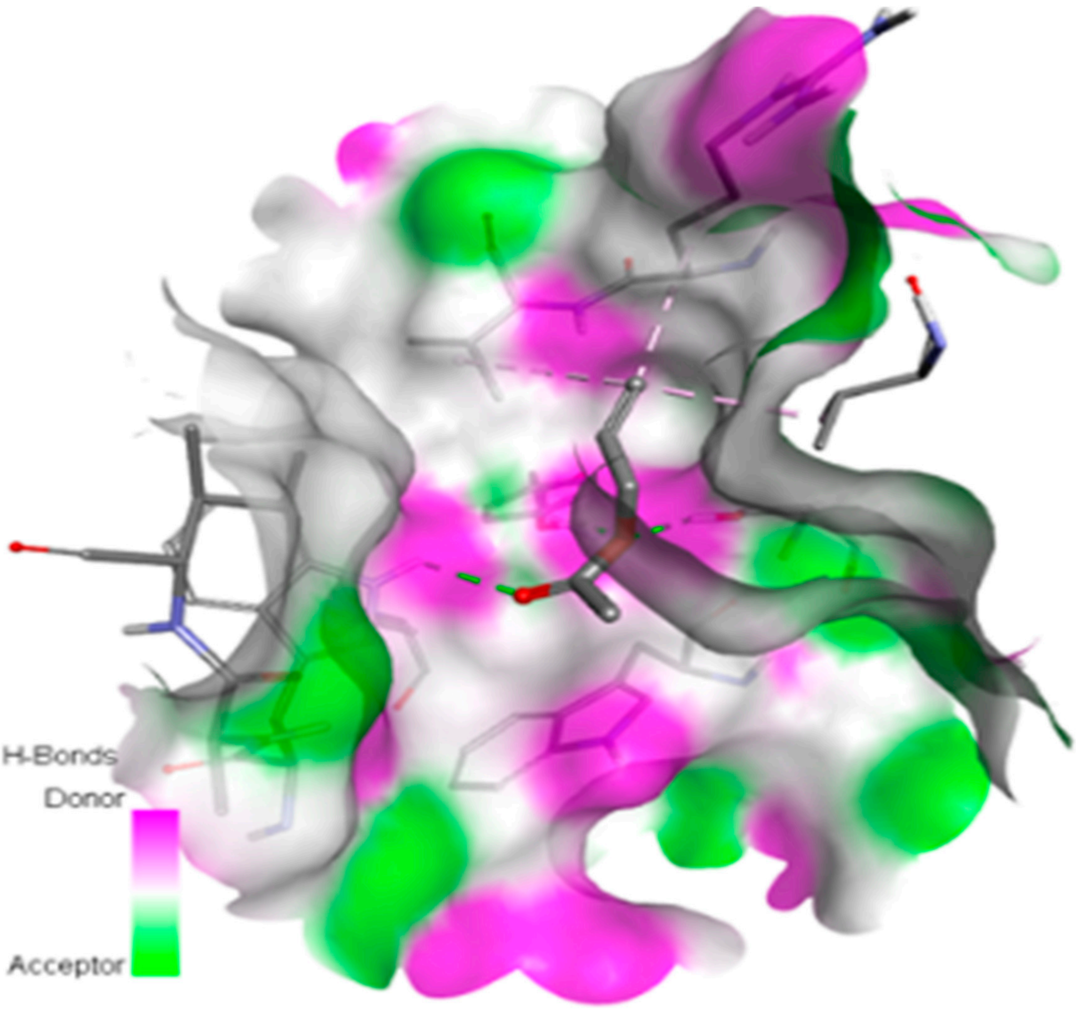

B) Allyl acetate

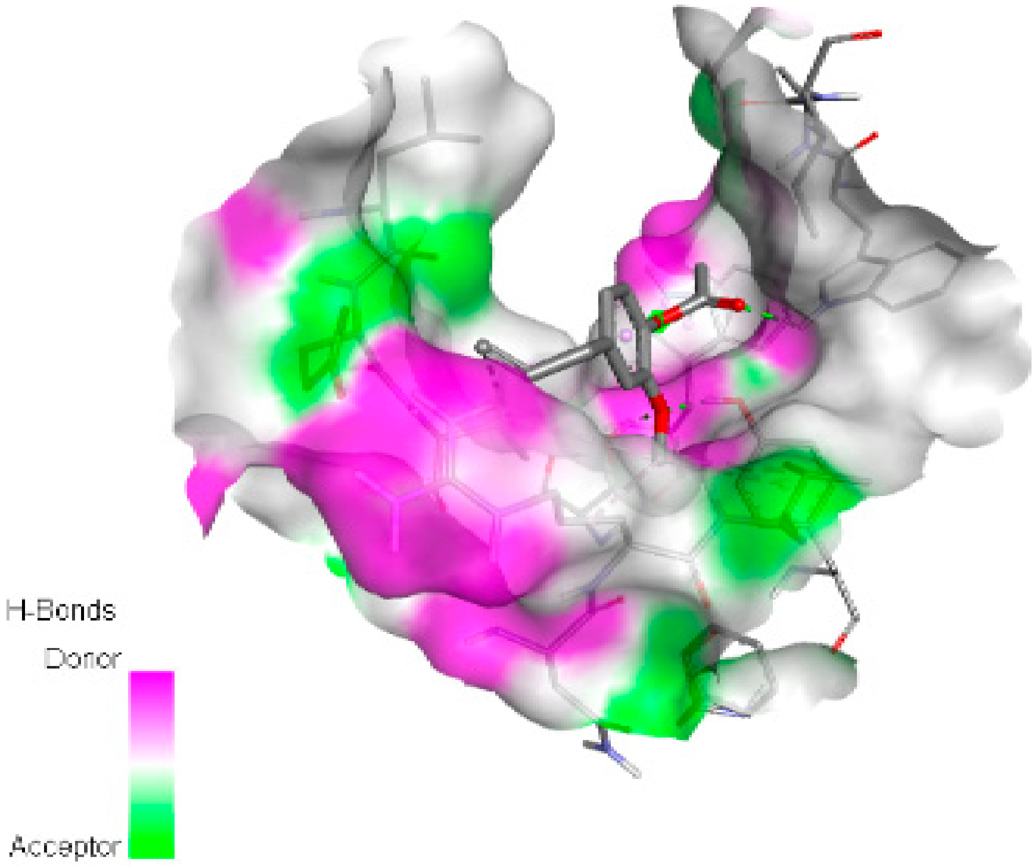

C) Kresoxim methyl

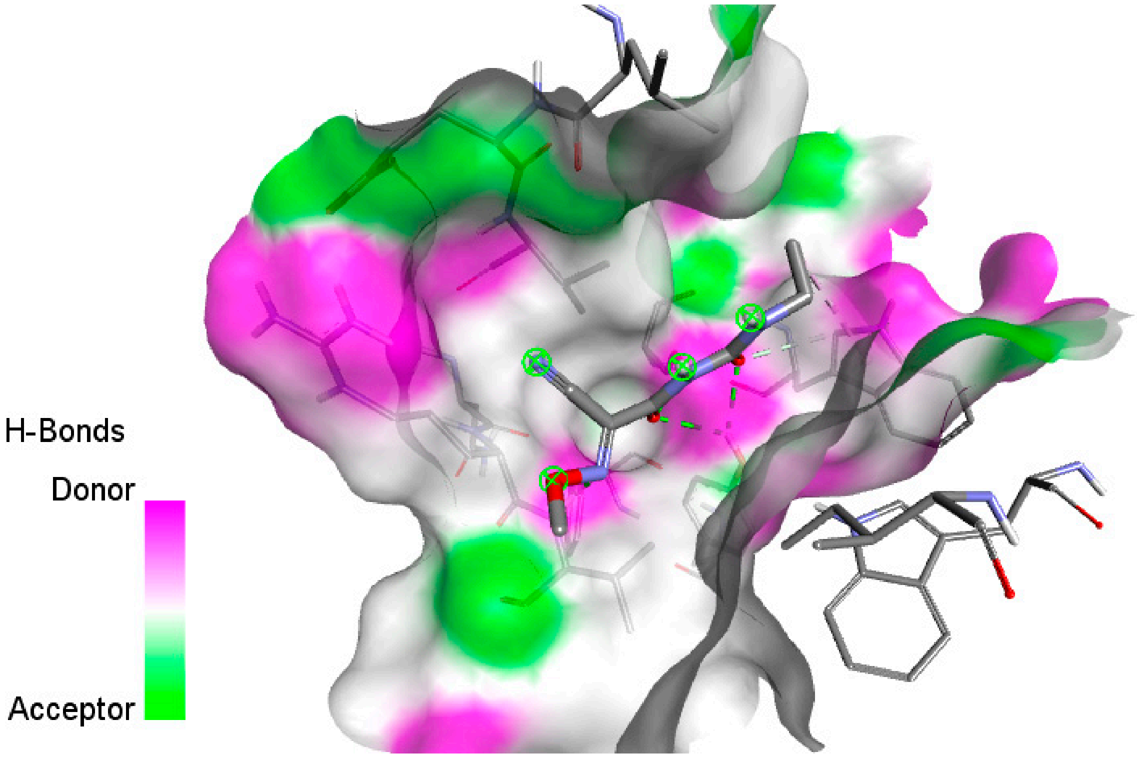

D) Curzate
