# Supplementary material for: Identification of Potential Phytochemical/Antimicrobial Agents against Pseudoperonospora cubensis Causing Downy Mildew in Cucumber through In-Silico Docking
Source: Plants (Basel). 2023 Jun 2;12(11):2202. doi: 10.3390/plants12112202 (PMC10255482; doi:10.3390/plants12112202)

Supplementary Figure S5. 2D visualization of the interaction between QNE 4 effector protein with top five phytochemicals A) Cucumerin A B) Cucumerin B C) Isocarpin D) Apigenin -7-*O*- glucosideE) Cucurbitacin-BF) Cucurbitacin-D G) Cucurbitacin-A H) Cucurbitacin-E I) Cucurbitacin-I

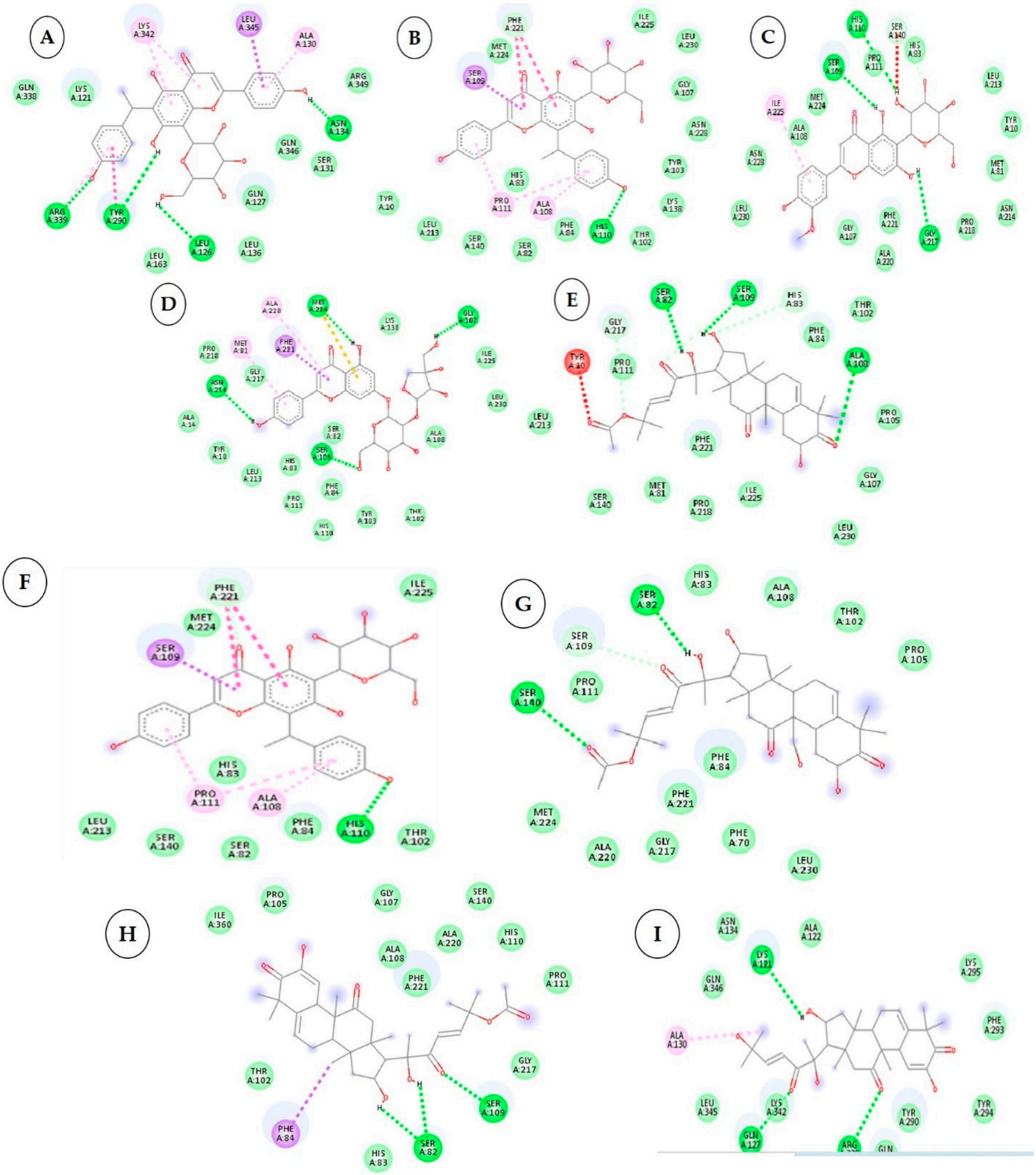

Supplement: Supplementary file 1 [file plants-12-02202-s001.zip › Supplementary Figure S5.pdf]
