# Supplementary material for: Identification of Potential Phytochemical/Antimicrobial Agents against Pseudoperonospora cubensis Causing Downy Mildew in Cucumber through In-Silico Docking
Source: Plants (Basel). 2023 Jun 2;12(11):2202. doi: 10.3390/plants12112202 (PMC10255482; doi:10.3390/plants12112202)

Supplementary Figure S6. 3D visualization of the interaction between cytochrome oxidase subunit 1 protein with top nine phytochemicals A) Cucurbitacin-I B) Saponin C) Cucurbitacin-D D) Cucurbitacin-E E) Swertianolin F) Cucurbitacin-A G) Cucurbitacin-B H) Cucumerin-A I) Luotonin A.

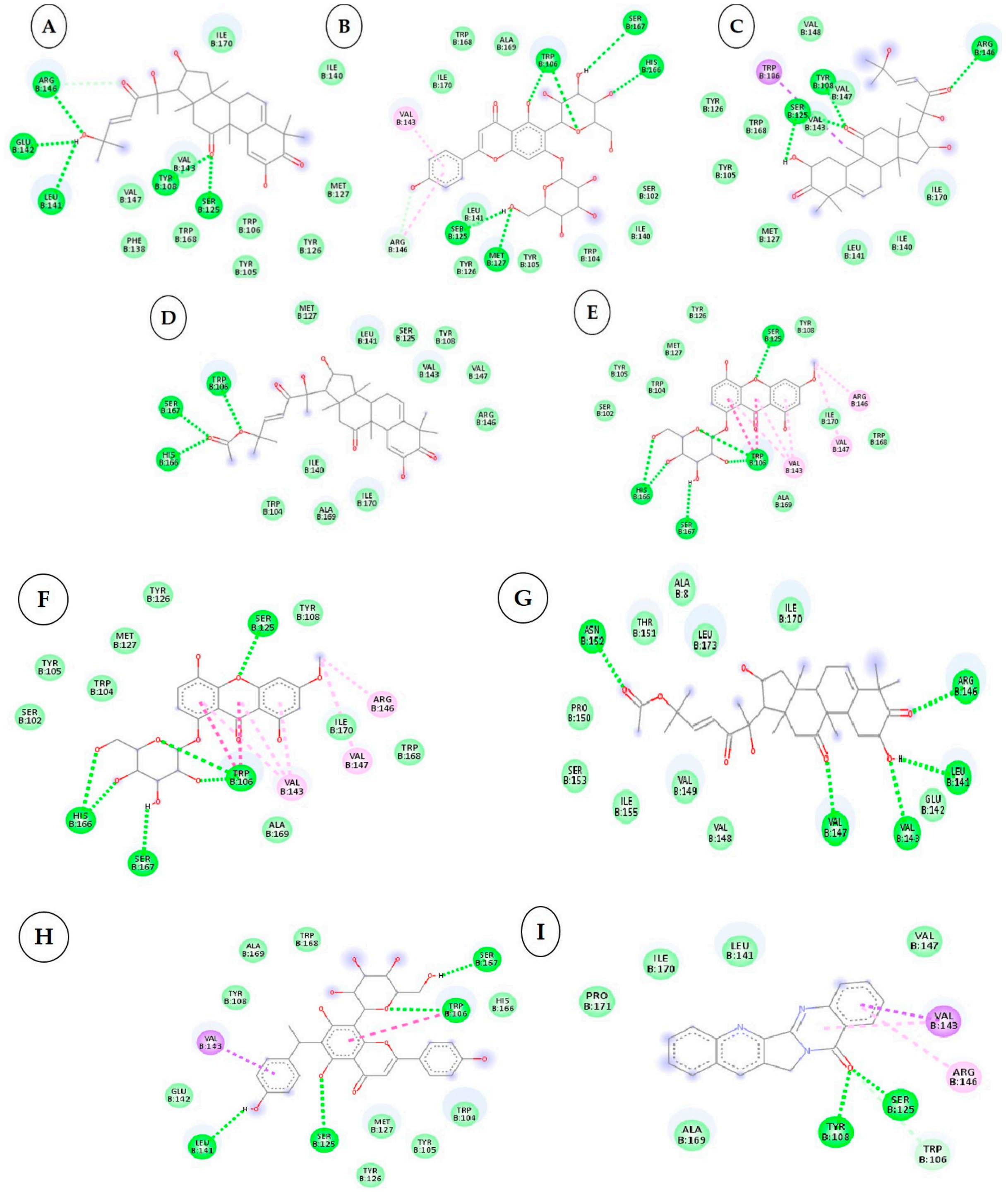

Supplement: Supplementary file 1 [file plants-12-02202-s001.zip › Supplementary Figure S6.pdf]
